# Understanding the work of general practitioners: a social science perspective on the context of medical decision making in primary care

**DOI:** 10.1186/1471-2296-9-12

**Published:** 2008-02-19

**Authors:** Robert Geneau, Pascale Lehoux, Raynald Pineault, Paul Lamarche

**Affiliations:** 1 Department of Family Medicine, University of Ottawa, Ottawa, Ontario, Canada; 2 Department of Health Administration, University of Montreal, Montreal, Quebec, Canada

## Abstract

**Background:**

The work of general practitioners (GPs) is increasingly being looked at from the perspective of the strategies and factors shaping it. This reflects the importance given to primary care services in health care system reform. However, the literature provides little insight into the medical decision-making processes in general practice. Our main objective was to better understand how organizational and environmental factors influence the work of GPs.

**Methods:**

We interviewed 28 GPs working in contrasting organizational settings and environments. The data analysis involved using structuration theory to enrich the interpretation of empirical material.

**Results:**

We identified four main factors that influence the practice of GPs: mode of remuneration, peer-to-peer interactions, patients' demands and the availability of other medical resources in the environment. These four conditions of action – what we call primary effects – can directly influence the performance of medical acts and time management, as well as the degree of specialization of GPs. Decisions related to each of those aspects can have a variety of both intentional and non-intentional consequences – what we call secondary effects – that are then likely to become conditions for subsequent action.

**Conclusion:**

This qualitative study helps shed light on the complex causal loops of interrelated factors that shape the work of GPs.

## Background

Primary care renewal is one of the cornerstones of current health care reform in industrialized countries. While a strong primary care system is associated with improved population health [1,2], there remains considerable debate about the economic impacts of primary care reform [3-5] and the cost-effectiveness of existing primary care funding and delivery models [6]. As a result, policy-makers, in an effort to get it "right", have been experimenting with a wide variety of strategies in order to align the work of primary care physicians with health system goals and objectives. They have introduced new primary care funding and delivery models [7,8] and promoted the development and dissemination of clinical practice guidelines [9].

These efforts all aim, through the use of either organizational or individual strategies, to improve medical decision making and quality of care. However, Blumenthal (2001) notes that improved medical decision making is as likely to increase expenditures for underused services as it is to reduce expenditures for overused services. Research into medical decision making in primary care has thus come to a crossroads: it is now under scrutiny for both cost and quality of care issues. Interest in this topic is fuelled by research showing there is still significant variation in the delivery of primary care services for similar medical conditions [10]. Such variation is observed in several aspects of physician practice, including diagnostic tests [11], patient referral to specialists [12], drug prescription rates, and frequency and timing of follow-up visits [13].

One of the current challenges in the field of primary care research is to explain such variation. Existing studies on practice variation suggest that individual, organizational and institutional factors all play a role. Physician characteristics such as age and sex [14], as well as personal values and psychological profile (e.g., risk averseness) have all been found to explain some of the variation [15,16]. GPs report often relying on their clinical intuition when dealing with the challenges and complexity of daily practice [17-19], sometimes more than on published practice guidelines [19,20]. Nonetheless, GPs also acknowledge being influenced by practice guidelines emanating from the professional system [21] and by their peers and colleagues [22]. Team work and organizational culture have also emerged as important explanatory factors [23,24]. There has been a vast body of literature focusing on the impact of payment mechanisms and financial incentives on GPs' practice and behaviours [25-27], with some studies concluding that they have significant effects on clinical decision making [28]. The availability of medical resources such as specialists or technologies in the surrounding environment can influence GPs as well [29,30]. Finally, GPs may vary in how they respond to patients' anxiety and to their requests for specific medical treatments or interventions [31,32].

Although the literature shows that all of these factors can influence the work of GPs, the "how" remains largely unexplained. Very few studies have attempted to paint the "whole picture" and to make explicit how individual physicians are connected to and influenced by their environment. Miller et al. [33] use complexity theory to explore the social and sensemaking dynamics within family medicine organizations. They conceptualize these organizations as complex adaptive systems composed of agents, including patients, office staff and physicians, who enact internal models of income generation, patient care and organizational operations. Such theorizations have led these researchers to suggest different strategies aimed at promoting change in practice and practitioner behaviour. Integrating theory into health services research is, we believe, a promising avenue in the quest to better understand the phenomena of clinical decision making and practice variation [34]. In this study, we consider the *praxis *of GPs as a social phenomenon and use concepts from Gidden's structuration theory [35] to better understand how clinical decision-making processes may be influenced by organizational and environmental factors. This theory shares similarities with complexity theory as they both focus on the production and reproduction of social systems. Gidden's work is far reaching and has inspired health researchers in multiple fields, including organizational science, nursing and information technology. One of the main strengths of structuration theory lies in its ability to integrate both individual (un)conscious needs and structural properties to explain why agents act the way they do. Using Gidden's main theoretical propositions about social actors, GPs' routines can be conceptualized as a continual flow of intentional actions bounded by (un)acknowledged conditions associated with individual and structural influences. For example, GPs' individual preferences about the content and organization of their clinical work may be viewed as partly driven by the quest for ontological security and self-esteem. Giddens defines ontological security as a *sense of safety *"expressing an autonomy of bodily control within predictable routines" (1984: 50). The structural influences that serve as conditions of action relate to rules and resources. Giddens has been criticized for not clarifying to what extent "structures" are comprised of virtual as opposed to actual resources [36]. For the purpose of this study, we broadly define rules and resources as the various supply and demand factors that can enable or constrain the work of GPs. In summary, structuration theory leads us to adopt two fundamental assumptions; 1) GPs cannot know or control all of the circumstances relevant to a specific action nor always precisely predict the consequences of their actions, and 2) every action creates the conditions for further action. Although this is an empirical paper, in the discussion we will show how these theoretical concepts contributed to data interpretation.

## Methods

We conducted a multiple case study between 2001 and 2003 in order to understand better how organizational factors influence the work of GPs in the Canadian province of Quebec. Case study methods involve an in-depth examination of a social phenomenon within its real-life context. [37] The case study approach has proven to be a valuable tool in health services research, especially when the boundaries between the relevant units of analysis are blurred [38]. In this study, we focus not on individuals, organizations or specific environments, but rather on the *relationships *between these different units of analysis.

### Selection of organizations and general practitioners

We used a stratified purposeful sampling strategy [39] to select eight primary care organizations. We selected four private clinics and four local community health centres (*Centres locals de services communautaires or CLSCs*). For each type of facility, we selected two located in a rural area and two in an urban area. GPs in private clinics are paid on a fee-for-service (FFS) basis, while GPs in CLSCs receive a salary. The objective was to select settings offering contrasting practice environments to GPs. This is consistent with theoretical research considering that the environment surrounding and permeating organizations can significantly impact on both organizational structure and individual behaviours [40]. In our study, the environment was considered an effect modifier of the relationships between organizational factors and professional practice. GPs working in private clinics accounted for approximately 70% of all primary care doctors in Quebec at the time of study, while approximately 13% of GPs worked in CLSCs. [41]. In 2002, a new model called "Family Health Groups" was implemented in the province (a reformed fee-for-service with financial resources allocated to practices for hiring nurses), but the FFS and CLSC models remain predominant. The most common model used in all Canadian provinces is the FFS model (70.4% of all primary care physicians) although health care is a provincial jurisdiction [42]. The core characteristics of the FFS and CLSC models are presented in Table 1.

**Table 1:** The CLSC and private clinic models

	**Private clinic**	**CLSC**
**Profile**	Oldest and predominant primary care delivery model in the province of Quebec (and in Canada). Physicians in Quebec are reimbursed by the Quebec Health Insurance Board- a public body established by the provincial government and reporting to the Minister of Health and Social Services.	Defined as a community and collaborative primary health care delivery model. CLSCs were introduced in the early 70s and receive their funding from the provincial government.
**Physician remuneration**	Fee-for-service (FFS).	Salary.
**Team composition**	Physician-based. Solo but predominantly group practice.	Multidisciplinary. Physicians are employees.
**Responsibility**	Patients	Population in a specific geographical catchment area.
**Profile**	Oldest and predominant primary care delivery model in the province of Quebec (and in Canada). Physicians in Quebec are reimbursed by the Quebec Health Insurance Board- a public body established by the provincial government and reporting to the Minister of Health and Social Services.	Defined as a community and collaborative primary health care delivery model. CLSCs were introduced in the early 70s and receive their funding from the provincial government.

We sent recruitment letters by mail to a small number of private clinics and CLSCs in Quebec. No solo practitioners were recruited because we wanted to study themes like organizational culture and professional collaboration. We interviewed a total of 28 GPs (17 men and 11 women): 23 from the original eight case study sites plus 5 recruited later from other settings in order to reach data saturation (we were unable to recruit additional physicians at the eight original sites, either because all had already participated or because some physicians declined to participate). In total, we interviewed 15 GPs working in CLSCs (9 in urban areas and 6 in rural areas) and 13 GPs working in private clinics (6 in urban areas and 7 in rural areas). Ethical approval for the study was obtained from the University of Montreal and informed consent was obtained before the interview.

### Data collection and analysis

We chose the semi-structured interview format in order to explore the influence of external factors on medical decision making and on professional life in general. The interviews lasted 90–180 minutes. We obtained ethics approval for our study from the University of Montreal, and the subjects signed an informed consent before the interview. The GPs were asked to freely elaborate on the different factors affecting their work. The interview guide also contained questions about the routines of GPs, such as how they manage their time (e.g., length of consultation) and how they interact with patients and colleagues. The interviews were tape-recorded and transcribed verbatim. The transcripts were then coded and analyzed using N6 software [43].

The coding scheme was developed gradually. We developed an initial list of codes informed by the literature on the behaviour of primary care organizations and physicians. We then analyzed the transcripts using an open-coding strategy in order to develop new categories of information and refine existing ones [44]. Ideas and categories generated through the line-by-line analysis were tested and further explored in subsequent interviews until saturation was reached. A final round of axial and selected coding was performed to add a conceptual layer to existing categories and subcategories and explore how they are interconnected [45]. We used the immersion/crystallization approach [46] as a transversal strategy, spending considerable time reading and absorbing the text in order to discover new meanings. This iterative analytical process also involves exploring and using contemporary social theories in order to (better) make sense of the data collected. We have found that concepts from structuration theory (Giddens, 1984) provide fertile insights into why GPs do what they do. We will elaborate more on the potential role of theoretical frameworks in health services research in our discussion.

## Results

The qualitative analysis revealed that the following organizational and environmental factors influence GP practice in various ways: mode of remuneration, peer-to-peer interactions, patients' demands and the availability of other medical resources in the environment. We first show the primary effects of these factors, that is how they influence three central aspects of GP practice: the medical decision-making process itself, time management, and the degree of specialization. We then explore the chain of intentional and unintentional consequences, or secondary effects, that flow from those primary effects.

### Primary effects on GPs' practice

**Figure 1:**
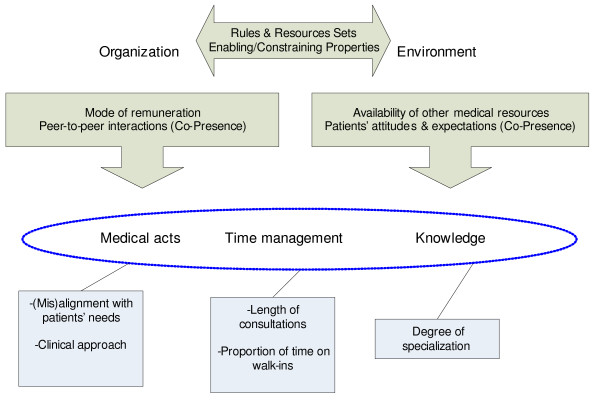
Primary Effects of Organizational and Environmental Factors.

Figure 1 summarizes the main findings. It shows the influence of organizational and environmental factors on GP practice. While there is a direct focus on medical decision making, the themes of time management and knowledge also emerge as central aspects of the GPs' professional life.

#### Mode of remuneration: understanding the rules of the game

Most of the respondents believe that the FFS mode of remuneration influences how GPs deliver services. The primary constraint of this mode of remuneration, according to FFS physicians, is that complex acts are under-remunerated:

"*There are some blatant injustices with FFS. I mean, it's more profitable to treat two cases of otitis than it is to treat one case of depression. It doesn't make sense. Bring on the otitis. Some GPs decide to do that*." (GP: private clinic, urban)

GPs who accept complicated cases can "maximize" their income by changing the way they handle routine health problems. For example, FFS physicians may over-examine patients with a routine health problem such as a cold or earache by systematically conducting unnecessary full examinations. In other words, the clinical decisions and clinical acts performed are not always based on patient needs:

"*A cold, the flu, gastroenteritis .... That's how we make money. I don't know a single GP who doesn't charge for a full exam for a cold or a case of the flu*." (GP: private clinic, urban)

*"For example, if a mother tells me, 'I think he's got an ear infection', if I only look at the ears, I get $10 but if I do a full exam, I get $30. I don't like to go at it this way, but at the end of the day I have to make money. I have debts." *(GP: private clinic, rural)

Conversely, physicians paid a salary feel they have a "purer" practice, since they do not have "to be concerned about billing codes when making a medical decision" (GP: CLSC, rural).

There was also a consensus among the interviewees that mode of remuneration influences the length of consultations. On average, GPs paid on a FFS basis took 10–15 minutes for a regular appointment, leading to what the FFS respondents called "treadmill pressure." In comparison, the average length of consultations for similar appointment types among salaried GPs was 20–45 minutes:

"*When I was working in a CLSC, I used to see 8 to 10 patients per half-day. In a private clinic you have office expenses; you schedule more patients because you know that 3 to 4 patients will cancel. You thus schedule them 15 minutes apart and you add 3 to 4 patients to the mix. I feel pressure when I work FFS*." (GP: private clinic, rural)

Another facet of time management affected by mode of remuneration is the proportion of time GPs devote to regular appointments versus walk-in services. In a community health centre, it is an organizational decision; salaried physicians rotate and spend a maximum of 6–7 hours per week seeing patients on a walk-in basis. FFS physicians tend to devote more time to walk-in consultations than do the salaried GPs: an average of 15-hours for the respondents in our study. Given the current cost structures, they feel this is the only way to make their practice "profitable." Devoting more time to walk-in consultations is also described as a way to "subsidize" appointment time slots devoted to time-consuming complex cases- this is the alternative to avoiding these complex cases altogether.

Finally, FFS physicians are also less inclined to participate in continuing medical education activities, primarily for financial reasons: "I think that if it's set in stone that you're not paid for it, you tend to disregard it over the long run" (GP: private clinic, urban).

#### The tacit influence of peer-to-peer interactions

All of the respondents indicated that they consider peer-to-peer interactions an important source of knowledge, which, in turn, is considered an important factor in the ability to maintain a "general" practice. However, many aspects of organizational life and structure can influence the effects of peer-to-peer interactions on the work of GPs. First, such interactions may simply not occur. Most FFS respondents described their practice setting as "solo in a group." The opportunities for discussing clinical cases, either formally or informally, are less frequent than in community health centres.

When GPs did experience peer-to-peer interactions on a regular basis, such interaction had two distinct effects: while they helped some GPs stay abreast of the latest medical discoveries and guidelines, they also motivated some GPs to stay within a particular professional "niche" in order to feel more secure:

"*Some physicians here are less comfortable with kids. Between us we know. I'm like that too. It's funny, I've done obstetrics for almost 20 years, but I've never put an IUD in. I ask my colleagues to do it. We help each other*." (GP: private clinic, rural)

A physician can join an organization in which GPs have all developed a "mini-specialty." From this perspective, the exclusion of certain groups of patients or diseases can be the unintended outcome of peer-to-peer interactions, rather than an entirely planned and intentional personal decision.

Peer-to-peer interactions also contribute to the establishment of certain norms around time management. Some perceive "fast" GPs, (6–8 patients per hour) as more hard-working, and more productive and effective. Some private clinic GPs work in an environment where slower physicians are perceived as "abnormal":

"*She was doing an excellent job as a doctor. But she was a major obsessive-compulsive .... For what I do in 15 minutes, she would take 45 minutes. She was unhappy with this type of organization. She left." *(GP: private clinic, rural)

#### Patients' demands: fighting the right battles

The issue of patients' demands influence physician behaviour was brought up by all respondents. It generated quite a diversity of responses.

First, GPs are not insensitive to patients' expectations and requests. Some patients insist on obtaining a medication, test or referral to a specialist. Some respondents, especially those paid on an FFS basis, said they do not always have the time or energy to explain why some tests or medications would not be useful. Moreover, the physician would then be dealing with a dissatisfied patient, one who would "probably persuade another physician to give him what he wants" (GP: private clinic, urban). For some physicians, it is a matter of "learning how to pick the right battles and differentiate between unnecessary treatments and those that are dangerous" (GP: private clinic, rural).

Secondly, many of the respondents mentioned that "waiting-room pressure" can affect how they manage their time. The patients' facial expressions when the physician steps briefly out of the office to call in the next person can lead to shorter consultations. One respondent reported how even the view of a full parking lot from his office window was enough to influence his work pace. The pressure for short consultations sometimes even comes from the patients themselves. Some patients have been "socialized" to be seen quickly; they remain standing or sit on the edge of their seat during the consultation:

"*When I started in this practice, some patients ... were not even sitting down .... Or they were already almost undressed before the door was open. I had to tell them, 'Sit down, I have to ask you some questions first.' But some preferred to remain standing; they were used to being seen in 5 minutes*." (GP: private clinic, rural)

Thirdly, patients may also have a preference with respect to physician gender, or perceive some GPs as having expertise in a specific domain. Patients specifically ask to see these physicians, and a "natural triage" takes place within the organization.

Finally, user characteristics have a major impact on both the length of consultations and the scope of practice of GPs. Some respondents deliberately avoid specific groups of patients:

"*There is a degree of uncertainty that I do not want to be dealing with at this stage in my career. If someone brings a baby into my office, I'm going to be sweating*." (GP: private clinic, urban)

Elderly people and those with mental health problems require much more time, and not all GPs are willing to take them in their practice. The search for a feeling of security in clinical routines has therefore led many physicians to develop "mini specialties," i.e., to orient their practice toward specific diseases or patient groups. According to them, it is important "to be good in a field you like" (GP: private clinic, urban). This requires finding strategies to help them better "control" the level of uncertainty in daily activities.

#### Availability of other medical resources in the environment: the impact of working in a rural area

For most of the rural respondents, the difficulty accessing specialized and technological resources means they need to adopt a more in-depth "clinical" approach before referring a patient. What was, at the outset, described as a constraint (limited options, source of uncertainty) was subsequently rationalized by some physicians as an opportunity to be "more evidence-based."

Physicians have no choice but to follow their patients up to the "limit" of their skills. Specialists also offer better support to physicians who have tried everything before referring a patient:

"*You only send your most complicated cases. You know, if you didn't investigate enough ... they end up knowing you. From the outset, it's tacit; they expect that a certain number of things have been tried and done by the general practitioner*." (GP: CLSC, rural)

GPs who practise in areas where there is a shortage of medical resources feel additional pressure to be up-to-date in as many fields as possible. They have more opportunities to put their knowledge into practice. The respondents from rural areas felt more "generalist" than their counterparts in urban practices. The need to learn is stimulated by exposure to diverse and complex health problems.

Rural GPs practising in under-serviced rural areas tend to accept new patients more regularly, even if they feel they have reached their full capacity. The large number of patients also forces them to keep up a fast consultation pace. The salaried GPs in our sample who practiced in rural under-serviced areas worked at a pace similar to that of FFS physicians. Rural FFS physicians spent on average 5 minutes longer in consultation than did their urban counterparts (20 minutes versus 15 minutes), while rural CLSC physicians had shorter consultations than did urban CLSC physicians.

### Secondary effects: the monitoring of unintended consequences

As we have seen, organizational and environmental characteristics influence three central aspects of GPs' work. These aspects can influence each other as well as other dimensions of primacy care practice. These secondary effects are often unintentional from the GP's perspective and, for some GPs, they may also be unacknowledged.

Time management is the cornerstone upon which the other aspects of a GP's practice are contingent. For example, as the proportion of time dedicated to walk-in services increases, the physician's availability for regular appointments decreases. This situation translates into a delay of sometimes 1–3 months between regular appointments, which, according to all respondents, is a source of uncertainty and anxiety:

"*I can tell you that it is very stressful to know that you can't see a patient ... within a month or two*." (GP: private clinic, urban)

GPs have developed various strategies to cope with this kind of situation, such as the creation of a "secret office" (periods over which the secretaries have no control), consultations during meal times and breaks, and giving patients more responsibility in identifying and monitoring their symptoms. However, a constant state of crisis and patient overflow also translates into shorter consultations, which in turn are associated with less communication between patient and physician, less emphasis on prevention and counselling, more medical uncertainty and, ultimately, more tests, prescriptions and referrals. For example, a difference of 5 minutes in the length of consultation is enough to modify a GP's approach and attitude. Under time constraints, there is an incentive to decrease communication. Several of the interviewees who had experienced both models of care over the course of their career acknowledged that they practice differently depending on the model under which they find themselves working. In a private clinic, the pressure for shorter consultations often leads to the "one problem per visit" rule:

*"There are some who work in two rooms. They open the door, they show no sign of friendliness that might encourage further discussion. When you are friendly, when you smile, when you are attentive, you get a second and a third question. That's for sure. And often a second or third problem .... What I mean to say is that you are a victim of the way you practise .... Here I have to tell my patients that I will deal with the most urgent problem first and leave the other less urgent complaints for subsequent visits." *(GP: private clinic, urban)

Shorter consultations also influence the level of completeness of case histories and how many questions will be asked by the physician. Ultimately, these two aspects also influence clinical decisions:

*"If you don't have the time to see your patient, you ask for tests. If you don't have the time to interview your patient, you ask for more tests." *(GP: private clinic, urban)

**Figure 2:**
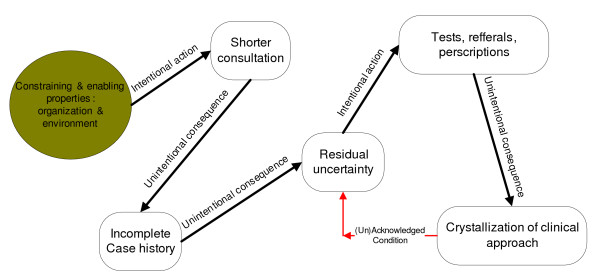
Secondary Effects of Structures on GPs' Work.

Figure 2 illustrates this domino effect of shorter consultations leading to less thorough case histories, then to more medical uncertainty, and finally to more tests, referrals and prescriptions. In the long run, it can lead to crystallized patterns and mindsets about how to practice medicine, and it comes to influence, indirectly, what GPs and patients expect and define as normal in a clinical encounter.

Furthermore, a strong emphasis on walk-in services can lead to the slow erosion of the skills and knowledge needed to manage chronic diseases:

*"And what is difficult to learn when you follow a diabetic or a hypertensive, for example, is how to schedule meetings, what you need to do, secondary prevention, how you should intervene. When you've been doing only walk-ins for ten years, you no longer know, or no longer want to know." *(GP: private clinic, urban)

Peer-to-peer interactions, themselves enabled or constrained by external factors, can also affect the GP's knowledge about specific fields and procedures. All in all, these relationships show that a variety of different aspects of a GP's practice overlap. It is a web of complex relationships where the intentional or unintentional consequences of an action then become the conditions for subsequent actions.

## Discussion

By looking at the work of GPs through the lens of theories aimed at explaining human action, this study contributes to opening the "black box" of medical decision making and professional practice in primary care. All studies focusing on practice variation and medical decision processes carry, or are based on, a specific set of assumptions about human behaviour and action. However, very few studies make these assumptions explicit; the "theory" remains invisible. While the role of theory in qualitative research is still being debated, several authors emphasize that such theory pertains to more than just methodology and underlying epistemologies [47]. Selecting and using specific theoretical concepts and constructs as a starting point or during the course of a study can enrich the interpretation of empirical material. In summary, theories, even grand theories, can be helpful in the fields of primary care and health services research, and researchers can benefit by exploring such theories and incorporating them into their research. In this study, structuration theory in particular has enriched our analytical perspective even though we have exploited only a fraction of Gidden's body of work.

The benefits of using theory to guide data analysis become apparent even when not engaging in full-blown theoretical research. For example, the concept of *ontological security – *defined as a sense of safety largely dependent on predictable routines – has rich implications for understanding the work of GPs. Our interviews with physicians revealed that developing a sense of security is one of the predominant facets and preoccupations shaping their professional experience and practice. GPs are partly driven by the desire to reduce the level of uncertainty in their daily activities, and each individual has his or her own degree of risk averseness. A study by Forrest et al. [48] has shown that primary care physicians with less tolerance to uncertainty tend to refer patients more often to specialists. With an increasingly complex clientele and rapidly evolving medical knowledge, interventions and prescription drugs, the respondents in our study expressed concerns about retaining enough control over their knowledge and skills. Other studies have also cast light on this phenomenon of insecurity in GPs. For example, in a survey of 1,276 Norwegian GPs, one-third stated they felt professionally vulnerable and ill-equipped to deal with rapidly changing knowledge [49], raising questions about the future of general medicine in health care systems [50]. Our study has highlighted some of the coping strategies and basic anxiety-controlling mechanisms adopted by GPs, including a preference for specialized practice, which is easier in an urban setting, or group practice, which enables the sharing of knowledge and clinical skills. This study also shows how GPs' choices and decisions are embedded in the wider socio-organizational context.

However, the need for predictable routines and an appropriate comfort zone and sense of security sometimes clashes with some of the structural properties of GPs' environment. While numerous studies have investigated, for example, the impact of mode of remuneration on GPs' behaviours, the original contribution of our study is its demonstration of the interconnectedness between different factors forming complex causal loops. This is where Gidden's work on the conditions of action and their (un)intentional consequences is particularly powerful. We have shown that depending on the particular mode of remuneration in place, intentional actions such as deciding on a specific appointment interval (e.g., every 10 or 15 minutes) can have unintentional consequences that can become the conditions for subsequent actions. For example, GPs must react to situations of clinical uncertainty that they themselves have helped to create. We have shown that for some GPs, medical uncertainty is sometimes the result of incomplete case histories or excessively long intervals between follow-up visits, which in turn can be linked to initial time management strategies. Time management is thus a central aspect of GP practice since it can trigger a domino effect on medical decision-making processes. For example, two studies concluded that shorter consultation times are associated with more antibiotic prescriptions and more laboratory tests [51,52]. Some evidence suggests that patients, especially those with complex and multiple problems, who seek help from a doctor who spends more time with them are more likely to have a consultation that includes important elements of care [53,54]. However, a systematic review that only considered controlled trials concluded there is insufficient evidence to support the claim that consultation length could be directly associated with observed differences in problem recognition, examination, prescribing, referral or investigation rates [55]. However, there was some evidence that blood pressure was checked more frequently and smoking discussed more often when more time was available. Finally, our study also shows that some GPs are influenced by patients' requests for tests and referrals, especially when time is a scarce resource. Patients' influence on health care delivery decisions is a phenomenon that has also been highlighted in other qualitative studies [31,56]. Our findings suggest that practicing in an environment where the availability of specialized and technological resources is limited has a counterbalancing effect.

The demonstration of secondary effects and causality loops is almost nonexistent in the literature on medical practice in primary care settings despite the known complexity of decision-making processes in family medicine. A study by Diwan, Sachs & Wahlstrom (1997) [57] showed that practice in primary care settings involves knowledge and attitude formation before new practices become crystallized. These types of analysis can help identify the key aspects of general practice that are most likely to influence and improve medical decision making.

## Conclusion

Individual, organizational and environmental factors make up a complex and dynamic whole. Our findings show that the structuration processes of GP practice involve various conditions of action that go beyond organizational structure and mechanisms. Policies and efforts aimed at influencing the work of GPs must take into consideration this complexity and the various sources of influence at play. Our findings suggest that time management is a crucial aspect of GP practice, one which serves as a background condition for a wide range of processes and decisions. The implications are numerous and challenging for decision makers. Further research could explore whether having more doctors working at a slower pace would be an effective cost-saving strategy for the health care system, even if the GP-population ratio must increase to ensure adequate access. This view challenges the current volume-centred definitions of "performance" and "productivity" at the primary care level, but it is grounded in the experience of general practitioners struggling to maintain professional principles in a complex and rapidly changing environment.

## Competing interests

The author(s) declare that they have no competing interests.

## Authors' contributions

RG carried out the case studies and conducted the interviews, led the data analysis process and drafted the manuscript. PL, RP and PL participated in the design of the study, assisted with data analysis and critically revised the manuscript for important intellectual content. All authors read and approved the final manuscript.

## Pre-publication history

The pre-publication history for this paper can be accessed here:


